# The effects of *Pandanus amaryllifolius* (Roxb.) leaf water extracts on fructose-induced metabolic syndrome rat model

**DOI:** 10.1186/s12906-019-2627-0

**Published:** 2019-08-28

**Authors:** Nur Hidayah Reshidan, Suhaila Abd Muid, Norshalizah Mamikutty

**Affiliations:** 1 Faculty of Medicine, Universiti Teknologi MARA, Cawangan Selangor, Kampus Sungai Buloh, Jalan Hospital, 47000 Sungai Buloh, Selangor Malaysia; 2 Institute of Pathology, Laboratory and Forensic Medicine (I-PPerForM), Universiti Teknologi MARA, Cawangan Selangor, Kampus Sungai Buloh, Jalan Hospital, 47000 Sungai Buloh, Selangor Malaysia; 3 Sulaiman Al Rajhi College, Faculty of Medicine, Kingdom of Saudi Arabia, Bukayriyah, 51941 Saudi Arabia

**Keywords:** Metabolic syndrome, *Pandanus amaryllifolius*, Pandan, Fructose, Obesity, Hypertension, Hyperglycaemia, Dyslipidaemia

## Abstract

**Background:**

Metabolic syndrome is a non-communicable disease inclusive of risk factors such as central obesity, hypertension, hyperglycaemia and dyslipidaemia. In this present study, we investigated the ability of *Pandanus amaryllifolius* (PA) leaf water extract to reverse the cluster of diseases in an established rat model induced by fructose in drinking water.

**Methods:**

Thirty healthy adult male Wistar rats (150–180 g) were randomly divided into three groups which included control (C; *n* = 6), PA extract (PAE; *n* = 6) and Metabolic Syndrome (MetS; *n* = 18). Food and fluid were given ad libitum for 8 weeks. These groups differed in fluid intake whereby rats received tap water, 10% of PA leaf water extracts and 20% of fructose in drinking water in group C, PAE and MetS, respectively. After 8 weeks, the MetS group was further subdivided into three subgroups namely MetS1 (*n* = 6), MetS2 (*n* = 6) and MetS3 (*n* = 6). The C, PAE and MetS1 were sacrificed. MetS1 group was sacrificed as the control for metabolic syndrome. MetS2 and MetS3 groups were treated with only tap water and 10% of PA leaf water extract respectively for another 8 weeks. The parameters for physiological and metabolic changes such as obesity, hypertension, hyperglycaemia, dyslipidaemia, and inflammatory biomarkers (NFκβ p65, TNFα, leptin and adiponectin) were measured.

**Results:**

The intake of 20% of fructose in drinking water induced full blown of metabolic syndrome symptoms, including obesity, hypertension, dyslipidaemia and hyperglycaemia in male Wistar rats. Subsequently, treatment with PA leaf water extract improved obesity parameters including BMI, abdominal adipose tissue deposition and adipocytes size, systolic and diastolic blood pressures, fasting plasma glucose, triglycerides, high density lipoprotein with neutral effects on inflammatory biomarkers.

**Conclusions:**

Administration of PA in metabolic syndrome rat model attenuates most of the metabolic syndrome symptoms as well as improves obesity. Therefore, PA which is rich in total flavonoids and total phenolic acids can be suggested as a useful dietary supplement to improve metabolic syndrome components induces by fructose.

## Introduction

Metabolic syndrome is a continuous growing problem consists of multiple metabolic dysfunctions cluster such as central obesity, hypertension, hyperglycaemia and dyslipidaemia that lead to cardiovascular diseases (CVD) [1], diabetes and stroke [2]. The common pathways gives domino effects to the pathogenesis metabolic syndrome in humans [3]. Metabolic syndrome was defined by International Diabetes Federation (IDF), National Cholesterol Expert Program Adult Treatment Program III (NCEP ATP III), World Health Organization (WHO) [4] and harmonized criteria which includes central obesity, elevated triglyceride (TG), reduced high density lipoprotein (HDL), raised blood pressure (BP) and fasting plasma glucose (FPG) [4]. Generally, metabolic syndrome is diagnosed when someone has central obesity with any of two of the risk factors [5]. Statistics revealed, 20–25% of adult population is affected by metabolic syndrome worldwide in developed and developing countries [6, 7]. The epidemic intake of refined carbohydrate such as fructose together with saturated fats increased the metabolic syndrome pandemic worldwide. In this study, the metabolic syndrome was experimentally induced in rats using 20% of fructose in drinking water as described in a previous study as it produced full blown of metabolic syndrome [7].

The aetiology of metabolic syndrome is a multi-factorial and involves environmental and genetic factors [8]. The mixture of modern sedentary lifestyle and diet rich in fat with lesser dietary fibre, bioactive ingredients and micronutrients enhances the progression of metabolic syndrome led to overweight, IR, hypertension and hypercholesterolemia [9, 10]. Thus, the prevention of metabolic syndrome is essential including weight loss, healthy diet, exercise, pharmacological treatment and bariatric surgery [11]. However, most of the available medications are sub-optimally effective and are associated with adverse drug reactions.

Natural products have previously shown efficacy in the treatment of metabolic syndrome related conditions such as chia seed [12], caffeine [9], ellagic acid [13], ferulic acid [14], purple carrot [15], green tea extract [16], olive extract [17], black current [18], quercetin [19], seaweed [20], and cocoa tea [1]. It is suggested that, Pandan (*Pandanus amaryllifolius* Roxb) could be beneficial for prevention of metabolic syndrome. It is an herbaceous plant that is classified as Pandanus genus in Pandanaceae family. It is commonly known as ‘pandan-mabango’ or fragrant screwpine [21]. It can be found in tropical countries like South Asia which produce more than 400 species and widely used as a folk medicine to energize body, reduce fever and relieve indigestion [22, 23]. In this study we used Pandan from Bachok, Kelantan, Malaysia as it has a high content of total flavonoid and total phenolic acids [24]. PA has been reported as a valuable herbs for the treatment of diabetes mellitus [25]. Pandan tea has been shown to significantly reduce postprandial blood sugar in human while aqueous and ethanolic pandan extracts were shown to stimulate insulin secretion in RINm5F cells and inhibit activity of alpha-glucosidase enzyme [26]. The PA leaf ethanol extract exert antidiabetic effects on streptozotocin-induced diabetic mice [27]. The predominant phytochemical constituents in pandan may be useful to prevent metabolic syndrome [24]. Although the phytochemical profile of PA has been reported, sparse information exists on the biochemistry and pharmacological activity of pandan tea or pandan drinking water especially in metabolic syndrome, or there is lack of studies on the effects of PA on improvement of metabolic syndrome components.

Therefore, the aim of this study is to induce metabolic syndrome in animal and to determine the effects of PA leaf water extract on the component of metabolic syndrome and inflammation biomarkers in the fructose fed metabolic syndrome rats. These rats showed symptoms of metabolic syndrome including obesity, hypertension, dyslipidaemia and hyperglycaemia. Particularly, we measured physiological and metabolic changes such as the percentage of total body weight gain, body mass index (BMI), abdominal circumference (AC), abdominal adipose tissues deposition, number and size of adipocytes, diastolic and systolic BP, fasting lipid profile (FLP), fasting plasma glucose (FPG) as well as the expression of inflammatory markers such as NFκβ p65, TNFα, leptin and adiponectin to assess the effects of PA against metabolic sydrome.

## Methods

### Animal and diets

Thirty healthy adult male Wistar rats with a body weight of 150–180 g were supplied by the Laboratory Animal Facility and Management (LAFAM), Universiti Teknologi MARA Puncak Alam. The rats were individually caged at Laboratory Animal Care Unit (LACU), Universiti Teknologi MARA Sungai Buloh. The rats were acclimatized to the housing facility for 14 days with free access to food and water prior to the experiment. The rats were housed in a controlled temperature (20 ± 22 °C), 12:12 h dark-light cycle with ad libitum access to food and specific fluid. The bedding was changed frequently. The experimental protocols were approved by Animal Ethics Committee Universiti Teknologi MARA (UiTM Care: 204/2017).

Rats were randomly divided into three groups including control (C; *n* = 6), PA extract (PAE; *n* = 6) and Metabolic Syndrome (MetS; *n* = 18). The number of animals was determined based on law of diminishing return. The rats were given tap water, 10% of PA leaf water extracts and 20% of fructose drinking water in groups C, PAE and MetS respectively. Food and fluid were given as ad libitum for 8 weeks. Following 8 weeks, the MetS group was further subdivided into three subgroups namely MetS1 (*n* = 6), MetS2 (*n* = 6) and MetS3 (*n* = 6), each consisting of 6 rats. The C, PAE and MetS1 were sacrificed. MetS1 group was sacrificed as the control for metabolic syndrome. MetS2 and MetS3 groups were treated with only tap water and 10% of PA leaf water extract, respectively, for another 8 weeks.

### Preparation of fructose drinking water (FDW)

The D-Fructose > 99% (Syarikat Systerm Malaysia) was used in this study to develop a rat model of metabolic sydrome. FDW was freshly prepared every alternate day. To prepare 20% of FDW, 20 g of fructose was diluted in 100 ml of tap water [7]. The water bottles were covered with aluminium foil to prevent fermentation. The FDW was given every day for 8 weeks as ad libitum to the rats to induce metabolic syndrome.

### Plant material

*Pandanus amaryllifolius* leaves were collected from Bachok, Kelantan. The voucher specimen number for this pandan leaves were MDI12818 and was approved by Dr. Mohd Norfaizal bin Ghazalli from Centre of Genebank and Seeds, Malaysian Agriculture Research and Development Institute (MARDI). The collection record was registered and kept at the herbarium in MARDI. Fresh pandan leaves were used for extraction in this study.

### Preparation of water extract of Pandanus amaryllifolius (PA)

The fresh pandan leaves were cut into small pieces of around 1.0–1.5 cm, washed with distilled water and air dried for 7 to 10 days. The small pieces of pandan leaves then were further placed in the oven at 45 °C till completely dry. Then, the leaves were powdered using mechanical grinder [24]. The extract was freshly prepared every alternate day by soaking and boiling 10 g of dried pandan powder in 100 ml water at 90 °C for 15 min [26]. The extract was then filtered with Whatman filter paper no. 1 before being administered to the rats ad libitum [26].

### Physiological measurement

Daily food, fluid and calorie intake was monitored for the entire experiment period. The daily food and fluid intake were measured by subtracting the remaining amount left in the cage from the initial amount provided [28]. The food intake was measured using an electronic weighing scale while fluid intake was measured using measuring cylinder. The calorie intake was calculated based on the amount of food and fluid intake. One gram of standard rat chow, fructose and pandan leaves contributes to 2.8 Kcal [7], 4 Kcal and 2.4 Kcal [29] respectively.

### Estimation of obesity related parameters

The percentage of total body weight gain, BMI and AC were measured as indicators of obesity risk factor [7]. Body weight was recorded weekly by using an electronic weighing scale. The increment in body weight was calculated by subtracting the final body weight to the initial total body weight of the animal and the percentage of total body weight gain was calculated [7]. BMI and AC were measured at the baseline, week 8 and at the end of the experiment. The BMI was calculated by dividing the weight (g) by the length (cm^2^) [30]. The length of the rats was measured between nasal and anal region [17]. AC were measured using a standard measuring tape around the anterior abdomen in centimetre (cm) [30]. The measurements were done under light anaesthesia using diethyl ether.

### Blood pressure measurements

BP was measured using the tail-cuff method with sphygmomanometer technique (Power Lab) at baseline, 8th and 16th week [7]. The instrument used was CODA mouse and rat tail system blood pressure by Kent Scientific. Before the BP measurement was taken, the rats were anesthetized by the inhalation of diethyl ether [31]. The anesthetized rats were placed in the holder and the animal tail was made to extend out of the rear of the holder. The Occlusion Cuff (O-Cuff) and VPR Cuff were installed at the CODA controller at O-Cuff and VPR ports respectively. Then, the O-Cuff was placed following with VPR cuff on the animal tail. Three readings were taken continuously [7]. Then, the average reading was calculated and taken as the final reading.

### Blood biochemistry

Blood samples were collected at the baseline, 8 and 16 weeks via orbital vein of anesthetized rats [7]. Prior to blood taking, the rats were fasted overnight and supplemented with tap water [17]. The drinking water in PAE, MetS1, and MetS3 groups were replaced with tap water before the blood collection was done. The blood samples were collected into blood collection tubes such as plain, K_2_EDTA and Potassium Oxalate tubes. Then, blood collection tubes were centrifuged at 1000x g and plasma was transferred in microcentrifuge tubes. The plasma was sent for FLP and FPG analysis at Pathology and Clinical Laboratory (M) Sdn Bhd. Plasma samples for adipokines and inflammation markers analysis were kept frozen at -80 °C.

### Protein expression

Activities of plasma and analyte concentrations were determined using enzyme-linked immunosorbent assay (ELISA) commercial kits according to manufacturer protocols [9, 32]. The NFκβ p65 was determined using rat NFκβ p65 ELISA kit by Elabscience, China. The TNFα, leptin and adiponectin were determined using rat TNFα Quantikine ELISA kit, Leptin Quantikine ELISA kit and Adiponectin Quantikine ELISA kit respectively by R&D Systems, USA. The analyte concentrations were analysed using Perkin Elmer 2030 Multilabel Reader Victor™ X5.

### Gross and microscopic changes of adipose tissue

Histological evaluation was performed on adipose tissues samples collected from the rats. The rats were anesthetized using diethyl ether [33] and euthanized by decapitation method. A longitudinal incision was made at the anterior aspect. The deposition of abdominal adipose tissues including omental, retroperitoneal and epididymal fats were observed in situ and then, the adipose tissues were removed [7]. The retroperitoneal deposition was defined as adipose tissue behind the kidney, along the back of abdomen [32]. The adipose tissues were washed with normal saline, weighed and dapped with gauze. The weight of adipose tissues was normalized to tibial length and expressed as milligram per millimetre mm)/mm) at the time of their removal [13].

Immediately after removal, the adipose tissues were fixed in 10% buffered formalin for 3 days. The formalin solutions were changed every day to remove traces of blood from the adipose tissues [17]. These tissue samples were processed using automate tissue processing machine. The samples were then dehydrated and embedded in paraffin wax. Thin sections of 5um were obtained and stained with Harris Haematoxylin and Eosin (H&E) and DPX mounted [7]. Histomorphometry of adipocytes was analysed using Cell D software (Olympus Soft Imaging Solutions GmbH). For the microscopic examination, three areas of 350 um X 250 um were used in each of the specimens. Three random, nonoverlapping fields per slide were taken to avoid biased analysis [12]. The adipocytes were counted only in the measuring areas and the cells at the border were left out [7]. The adipocytes size was estimated measuring the area, perimeter and diameter. The analysis was done in double blinded fashion.

### Statistical analysis

Data were analysed using SPSS version 24.0. Distribution of data (*p* value > 0.05) was determined by Shapiro-Wilk test. Normally distributed data were expressed as mean ± SEM. Skewed data were expressed as median with 95% of confidence interval (CI). The comparison of treatment timeline in the same groups was performed using Paired T-test. While for comparison between the two groups was performed using Independent T-test. Comparison between more than two groups were performed by one-way ANOVA. The criterion for statistical significance was *p* < 0.05. Post Hoc analysis was performed when the ANOVA test comes out significant.

## Results

### The effect of P. amaryllifolius on physiological changes

The effect of PA on dietary intake including food intake, food calorie intake, fluid intake, fluid calorie intake and total calorie intake were summarized in the Table 1. Despite lower food intake, the calorie intake was higher in MetS1 compared to C and PAE after 8 weeks due to higher fructose intake. Fructose has the highest calories to be compared with tap water and PA extract. The high calorie intake increased body weight, BMI, AC and abdominal adipose tissue deposition. Throughout 16 weeks, both MetS2 and MetS3 increased in food intake but decreased in fluid intake. Thus, the reduction in calorie intake leads to lower body weight, body weight gain, BMI, AC, and abdominal adipose tissue deposition.

**Table 1:** Dietary Intake for C, PAE, MetS1, MetS2 and MetS3 at week 8 and 16

Variables	C	PAE	MetS1	MetS2		MetS3	
	8th	8th	8th	8th	16th	8th	16th
Food intake (g/day)	27.26 ± 1.09	27.81 ± 0.77	19.85 ± 0.25^ab^	21.02 ± 3.40	26.90 ± 0.87*	21.50 ± 1.39	26.75 ± 0.47*
Food calorie intake (Kcal/day)	76.80 ± 2.82	77.88 ± 2.17	55.58 ± 0.714^ab^	58.86 ± 3.89	75.31 ± 2.42*	60.20 ± 0.99	74.90 ± 1.31*
Fluid intake (ml/day)	32.14 ± 1.08	36.83 ± 1.02	50.40 ± 1.98^ab^	47.11 ± 4.37	35.00 ± 2.82*	39.73 ± 1.22	34.42 ± 1.65*
Fluid calorie intake (Kcal/day)	0.00 ± 0.00	0.62 ± 0.17	281.67 ± 11.65^ab^	263.12 ± 24.45	0.00 ± 0.00*	222.31 ± 6.97	0.58 ± 0.28^c^*
Total calorie (Kcal/day)	76.79 ± 2.82	78.50 ± 2.12	337.24 ± 11.78^ab^	321.98 ± 24.51	75.31 ± 2.42*	282.51 ± 7.20	75.47 ± 1.31*

Note: Data are expressed as mean ± SEM (*n* = 6 for each group). ^a^*p* < 0.05 compared to C. ^b^*p* < 0.05 compared to PAE. ^c^*p* < 0.05 compared to MetS2. *p < 0.05 compared to 8th week

### The effects of P. amaryllifolius on metabolic changes

#### Obesity parameters

The effects of PA on obesity parameters including percentage of body weight gain, total abdominal fat deposition, BMI and AC were summarized in Table 2. The higher total calorie intake in MetS groups lead to higher percentage in body weight gain, BMI and AC after 8 weeks. The increased in total abdominal fat deposition including omental, retroperitoneal and epididymal fats indicated by increases in BMI and AC. Body weight gain were lowered with PA supplementation in PAE and MetS3 with lower total abdominal fat deposition.

**Table 2:** Obesity Parameters for C, PAE, MetS1, MetS2 and MetS3 at baseline, week 8 and 16

Variables	C		PAE		MetS1		MetS2			MetS3		
	0th	8th	0th	8th	0th	8th	0th	8th	16th	0th	8th	16th
Body weight (g)	248.5 ± 3.7	464.0 ± 13.8*	238.3 ± 5.7	448.2 ± 8.9*	243.5 ± 4.3	460.7 ± 13.8*	246.2 ± 3.6	480.2 ± 21.01*	547.7 ± 3.3^#^	234.7 ± 3.3	455.2 ± 12.8*	492.7 ± 11.7^#a^
Body weight gain (%)	–	45.37 ± 4.39	–	46.67 ± 1.79		48.38 ± 2.29	–	47.46 ± 1.52	11.91 ± 1.15	–	48.32 ± 1.41	8.16 ± 1.33^a^
Body Mass Index (g/cm^2^)	0.608 ± 0.02	0.790 ± 0.02*	0.638 ± 0.01	0.720 ± 0.02*	0.606 ± 0.01	0.783 ± 0.02*	0.583 ± 0.02	0.788 ± 0.02*	0.803 ± 0.01	0.561 ± 0.01	0.822 ± 0.05*	0.766 ± 0.04^#^
Abdominal Circumference (cm)	16.67 ± 0.31	21.08 ± 0.15*	17.00 ± 0.34	20.67 ± 0.33*	16.83 ± 0.21	21.25 ± 0.42*	17.33 ± 0.27	21.25 ± 0.38*	22.50 ± 0.18^#b^	16.91 ± 0.20	21.67 ± 0.40*	21.83 ± 0.25^a^
Total Abdominal Fat (mg/mm tibial length)	–	182.21 ± 14.96	–	189.81 ± 12.93	–	226.35 ± 18.89	–	–	283.67 ± 27.63	–	–	231.77 ± 18.49

Note: Data are expressed as mean ± SEM (*n* = 6 for each group). ^a^*p* < 0.05 compared to MetS2. *p < 0.05 compared to 0th week. ^#^*p* < 0.05 compared to 8th week

#### Adipose tissue

**Fig. 1:**
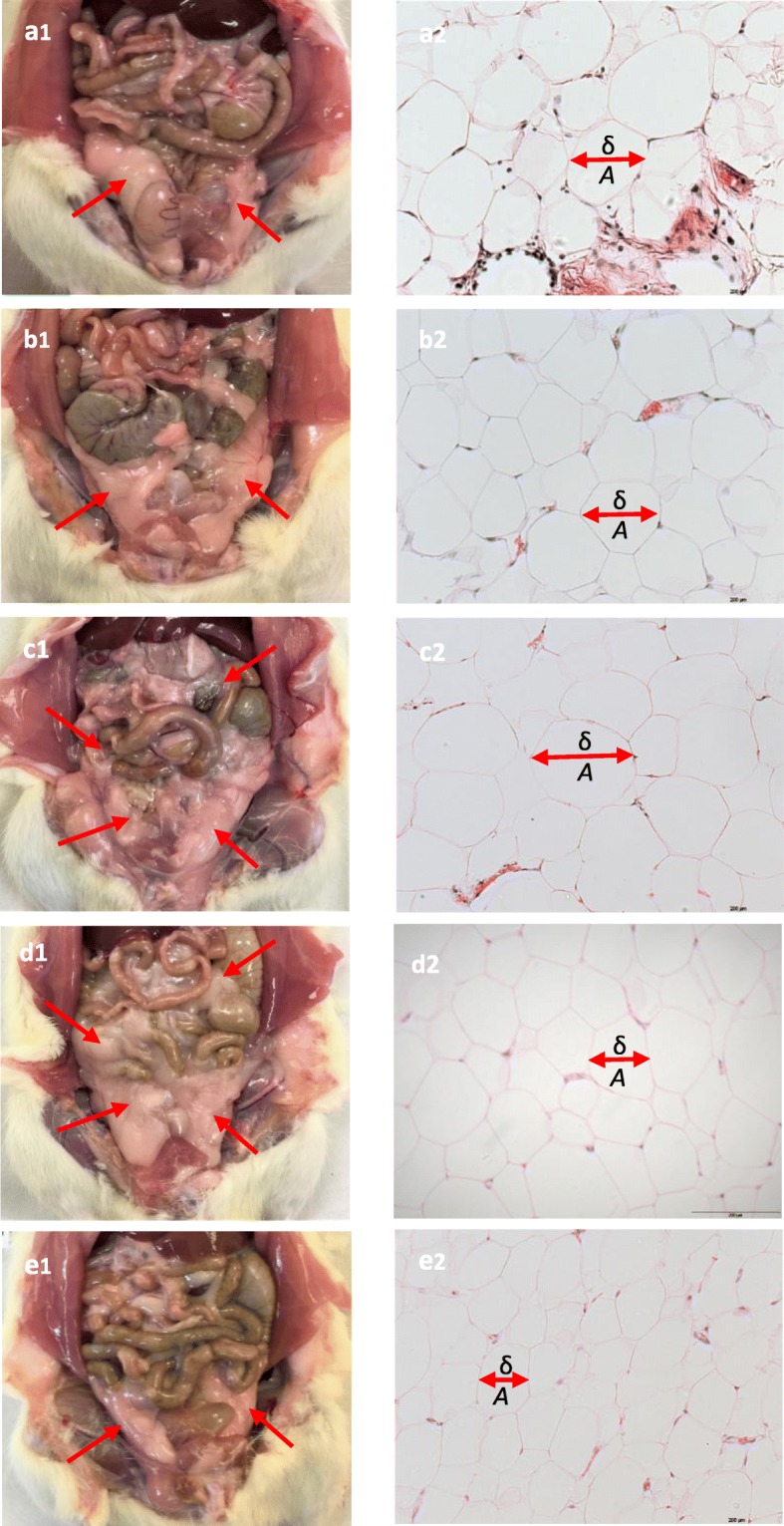
The abdominal adipose tissue deposition (**a1**, **b1**, **c1**, **d1**, **e1**) and the histomorphology (**a2**, **b2**, **c2**, **d2**, **e2**) of adipocytes of C, PAE, MetS1, MetS2 and MetS3 respectively. The deposition of abdominal adipose tissue was greater in MetS1 (**c1**) than C (**a1**) and PAE (**b1**) after 8 weeks of FDW consumption. The deposition of abdominal adipose tissue was lower in MetS3 (**e1**) than MetS2 (**d1**) with PA consumption. The histomorphology of adipocyte of MetS1 is different as compared to C and PAE where the size of adipocytes is increased in MetS1 as compared to C and PAE. The size of adipocyte which is indicated by diameter, perimeter, and area and the number of adipocytes was calculated. *A*: adipocyte, δ: diameter

The effects of in-situ abdominal adipose tissue deposition and the histomorphology of adipocytes are shown as in Fig. 1. The red arrow shows omental, retroperitoneal and epidydimal adipose tissue deposition. The in-situ observation and weight of abdominal adipose tissues deposition were higher in MetS1 than C and PAE after 8 weeks. Meanwhile, MetS3 showed the least abdominal adipose tissue deposition as seen by in-situ observation and lowered weight of abdominal adipose tissue deposition (Fig. 1e1) than MetS2 after week 16. There was no significant difference between MetS2 and MetS3 in abdominal adipose tissue deposition. MetS2 and MetS3 showed 20.2 and 2.33% higher of abdominal adipose tissue weight deposition, respectively compared to MetS1.

**Fig. 2:**
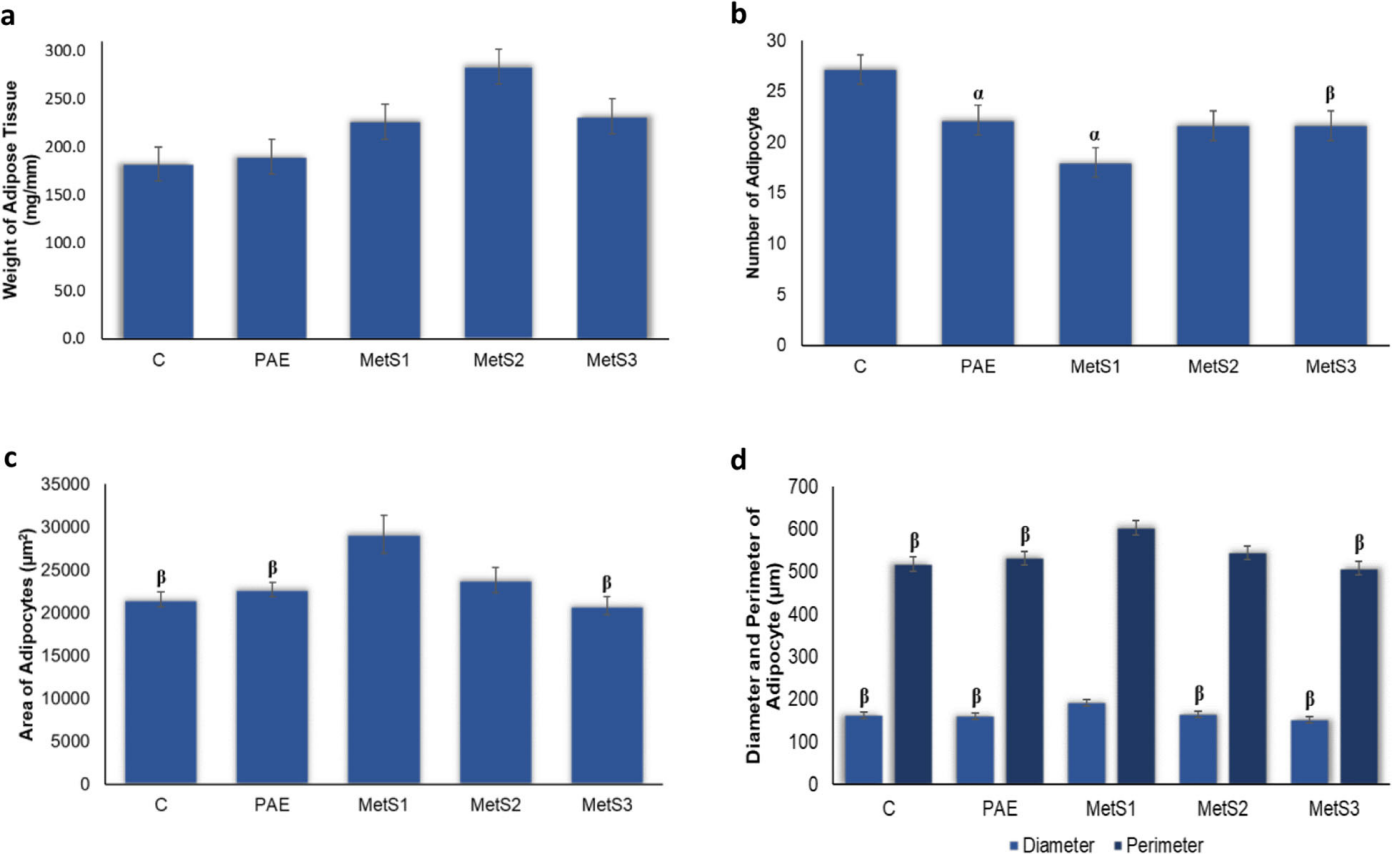
Adipose tissue parameters on **a** weight of adipose tissue, **b** number of adipocytes, **c** area of adipocytes and **d** diameter and perimeter of adipocytes in C, PAE, MetS1, MetS2 and MetS3. *n* = 6. ^α^*p* < 0.05 compared to C. ^β^*p* < 0.05 compared to MetS1

The effects of PA on histomorphology of adipocytes were determined through histomorphometry analysis including number, area, diameter and perimeter of adipocytes (Fig. 2). Histological findings revealed that hypertrophy in MetS1 as compared to C and PAE with lower number of adipocytes (Fig. 2b). The size of adipocytes was significantly higher in area, perimeter and diameter compared to C and PAE as shown in Fig. 2c and d. The number of adipocytes were increased in both MetS2 and MetS3 compared to MetS1but only significance in MetS3. The number of adipocytes ranged from 18 to 27. The trends in Fig. 2c showed that MetS3 has greater reduction compared to MetS1 in area of adipocytes with the PA supplementation. Meanwhile, the diameter and perimeter (Fig. 2d) were lower in MetS3 than MetS2 after 16 weeks. Figure 2d shows that the diameter ranges from 151.98 ± 13.21 μm to 192.14 ± 5.50 μm and the perimeter ranges from 508.91 ± 13.21 μm to 602.72 ± 22.0 μm. The lowest number of adipocytes reflects the highest in the area, diameter and perimeter of adipocytes.

#### Blood pressure

**Fig. 3:**
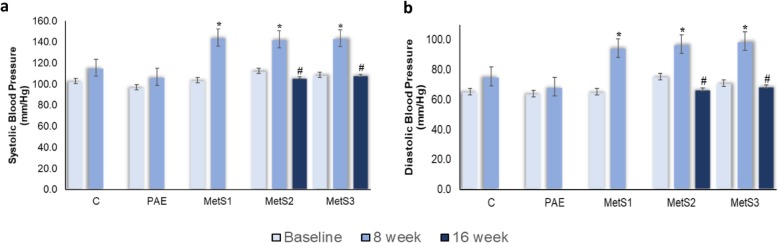
Blood pressure effects on **a** systolic and **b** diastolic BP in C, PAE, MetS1, MetS2 and MetS3. *p < 0.05 compared to 0th week. #*p* < 0.05 compared to 8th week

The effects of PA on systolic and diastolic BP are shown as in Fig. 3*.* There was no significant difference in systolic and diastolic BP at the baseline for C, PAE and MetS1. Systolic and diastolic BP was increased in MetS1 than in C and PAE after 8 weeks. The rats that consumed the most calories diet had developed high BP. The systolic and diastolic BP were also significantly increased in MetS2 and MetS3 after 8 weeks and these were normalized after week 16. However, there was no significant difference between MetS2 and MetS3 at 16 weeks.

#### Blood biochemistry

**Fig. 4:**
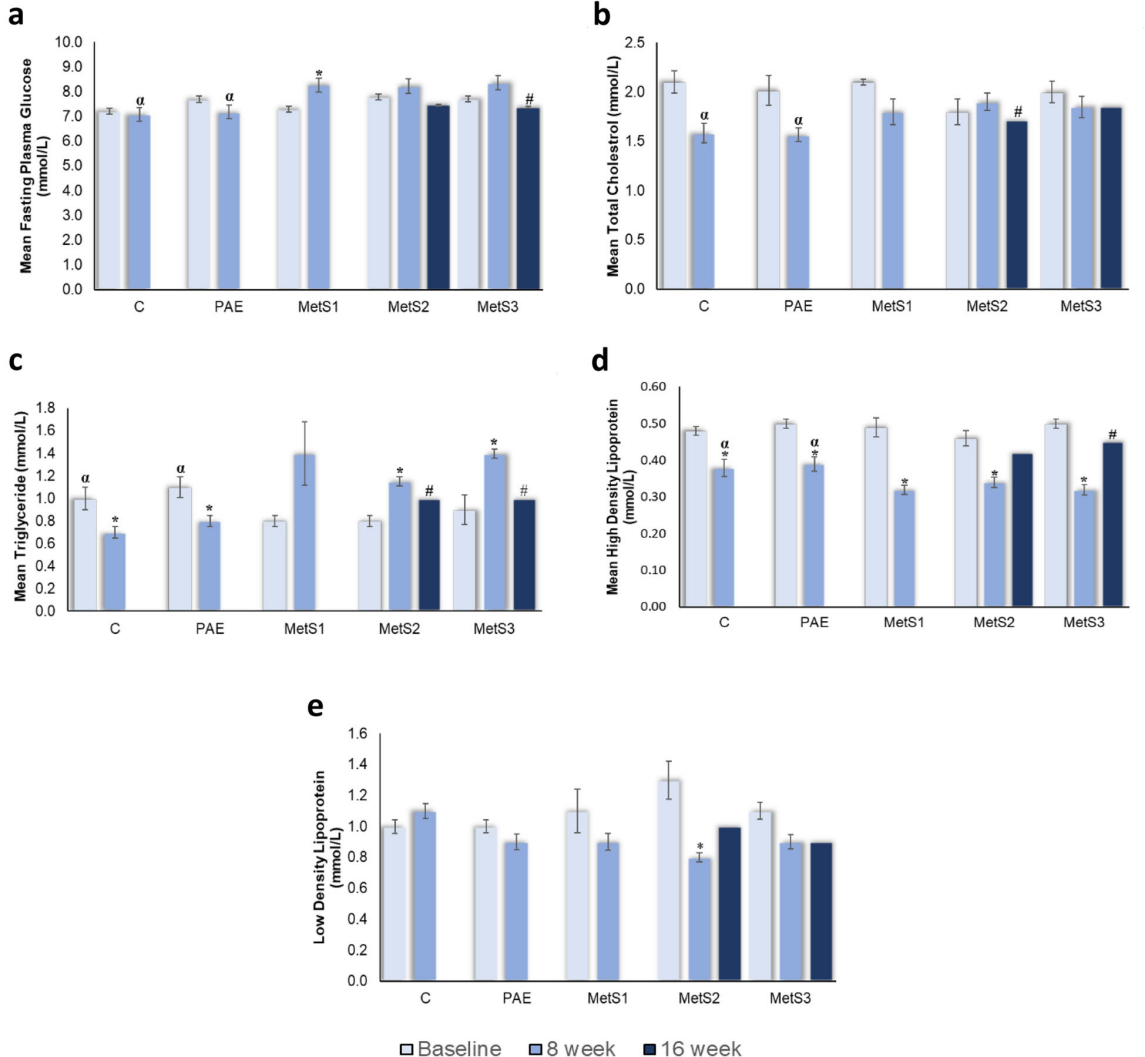
Blood biochemistry effects on **a** FPG, **b** TC, **c** TG, **d** HDL and **e** LDL in C, PAE, MetS1, MetS2 and MetS3. *n* = 6. *p < 0.05 compared to 0th week. #*p* < 0.05 compared to 8th week. ^α^*p* < 0.05 compared to MetS1

Figure 4 show the effects of PA on blood biochemistry including FPG, TC, TG, HDL and LDL. The effects of FPG were illustrated in Fig. 4a*.* The FPG levels ranged from 7.2 ± 0.17 to 8.4 ± 0.19 mmol/L. There was no significant difference of FPG level among C, PAE and MetS1 at baseline. The FPG remained unchanged in C, decreased in PAE and increased in MetS1 at 8 weeks. There was no significant difference in FPG level in MetS2 and MetS3 after 8 weeks. The inclined FPG level were reduced at week 16 but only significant in MetS3 with PA consumption.

Figure 4b shows the effects on total cholesterol (TC). The TC levels ranges from 1.5 ± 0.07 to 2.1 ± 0.11 mmol/L. There was no significant difference in TC level among C, PAE and MetS1 at baseline. The TC level was significantly higher in MetS1 than in C and PAE after 8 weeks. There was no significant difference in TC level between MetS2 and MetS3 after 8 weeks compared to baseline. MetS2 was significantly reduced and MetS3 remained unchanged in TC level at sixteenth week compared to eighth week with tap water and PA post supplementation respectively.

The level of TG was shown in Fig. 4c. The TG levels ranges from 0.70 ± 0.05 to 1.43 ± 0.28 mmol/L. MetS1 had lower TG level than C and PAE at baseline. The TG level were significantly reduced in C and PAE but increased in MetS1 after 8 weeks. The TG level was higher in Met3 than MetS2 after 8 weeks and significantly declined in MetS2 and MetS3 after week 16. However, MetS3 were highly reduced to 45.65% while MetS2 only reduced to 19.87% in TG levels as compared to eighth week.

Figure 4d shows the effects of HDL level. The HDL level ranges from 0.32 ± 0.01 to 0.50 ± 0.01 mmol/L. There was no significant difference among C, PAE and MetS1 at baseline. All C, PAE and MetS1 were significantly reduced after 8 weeks when compared to baseline. The HDL levels in MetS2 and MetS3 show no difference after 8 weeks of fructose induction and improved after 16 weeks but was significantly increased in MetS3 due to PA supplementation.

The effects of LDL level are illustrated in Fig. 4e. The LDL level ranges from 0.82 ± 0.03 to 1.30 ± 0.12 mmol/L. There was no significant difference in LDL levels among C, PAE and MetS1 at baseline. The LDL level was decreased in PAE and MetS1 but increased only in C after 8 weeks. The LDL level was decreased in MetS2 and MetS3 after 8 weeks compared to baseline. The LDL level in MetS2 was increased and MetS3 remain unchanged after 16 weeks. PA supplementation helps to sustain the LDL level in MetS3.

#### Protein expression

**Fig. 5:**
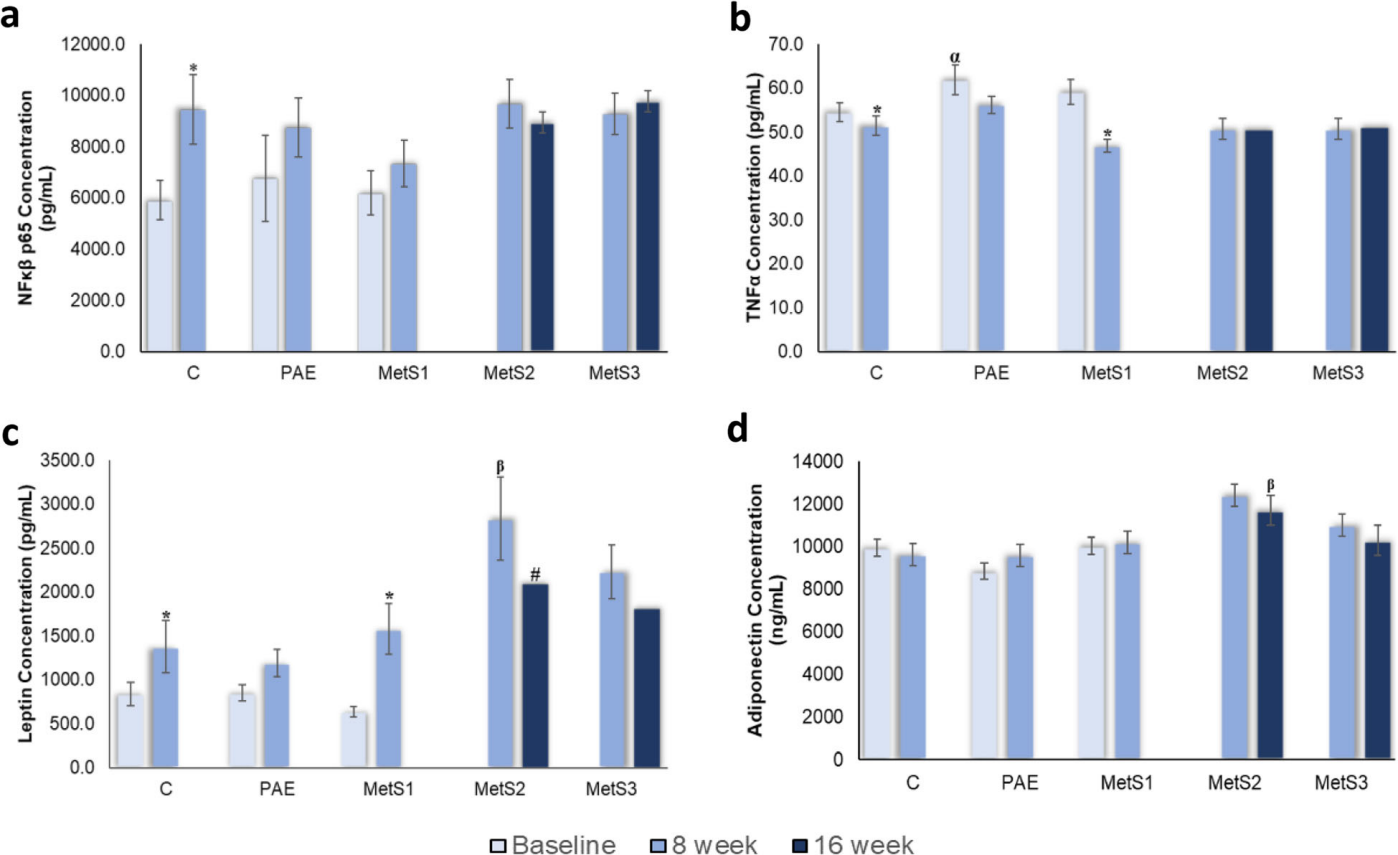
Protein Expression of **a** NFκβ p65, **b** TNFα, **c** Leptin and **d** Adiponectin in C, PAE, MetS1, MetS2 and MetS3. *n* = 6. *p < 0.05 compared to 0th week. #*p* < 0.05 compared to 8th week. ^α^*p* < 0.05 compared to MetS1. ^β^*p* < 0.05 compared to MetS3

Figure 5 shows the effect of PA on inflammatory protein level of NFκβ p65, TNFα, leptin and adiponectin. The expression of NFκβ p65 is shown in Fig. 5a. There was no significant difference of NFκβ p65 expression among C, PAE and MetS1 at baseline. The expression of NFκβ p65 was significantly increased in C after 8 weeks compared to baseline. There was no significant difference at eighth week and no significant difference after 16 weeks of NFκβ p65 expression in MetS2 and MetS3.

Figure 5b shows the expression of TNFα. The expression TNFα was higher in PAE than C and MetS1 at the baseline. The expression of TNFα significantly declined in C, PAE and MetS1 after 8 weeks compared to baseline. The expression of TNFα remain unchanged in MetS2 and MetS3 at 16 weeks compared to 8 weeks. However, there was also no changes of TNFα expression with the PA supplementation.

The effect of leptin expression is shown in the Fig. 5c. There was no significant difference of leptin expression among C, PAE and MetS1 at baseline. The leptin expression significantly increased in C and MetS1 at 8 weeks compared to baseline. MetS2 had higher leptin level than MetS3 at eighth week. Leptin level in MetS2 was significantly reduced at sixteenth week compared to eighth week. The MetS3 was also reduced at sixteenth week.

Figure 5d shows the adiponectin expression. There was no significant difference in the expression of adiponectin among C, PAE and MetS1 at baseline. There were no significant changes in the adiponectin expression among C, PAE and MetS1 at eighth week compared to baseline. MetS2 had higher adiponectin level than MetS3 at eighth week. There was no significant reduction of adiponectin level in MetS2 and MetS3 at sixteenth week when compared to eighth week.

## Discussion

The term metabolic syndrome has been recognised in the medical literature for more than 80 years [34, 35]. According to epidemiologic studies, the incidence of metabolic sydrome is increasing globally [9]. However, due to metabolic syndrome multifactorial causation, satisfactory experimental model to study the pathology of this syndrome are not available. Currently, many types of animal models and diet have been used to develop metabolic syndrome model. The choice of the metabolic syndrome model determines the experimental outcome to a great extent [36]. In this study, we have evaluated the therapeutic response of PA to attenuate features of metabolic syndrome in fructose induced rat model. Interestingly, the PA leaf water extract treatment was found to improve body weight gain, reduced BMI, less abdominal adipose tissues deposition, reduced adipocytes sizes in abdominal fat tissues, improve on systolic and diastolic BP, FPG, TG and HDL concentrations, accompanied by the differences of inflammatory protein levels of NFκβ p65, TNFα, leptin and adiponectin expression.

In this study, male Wistar rats fed with 20% fructose drinking water for 8 weeks to develop established metabolic syndrome animal model following previous study [7]. Following reversal protocol, the fructose induction was stopped once metabolic syndrome was completely developed and started with PA treatment. The rats presented all features of metabolic syndrome and particularly, obesity. These results have been previously characterised in details. An alarming metabolic syndrome prevalence can be seen with the high calorie intake of fructose [37]. The fructose sweet palatability increased the fluid intake and reduced the food intake in MetS groups (MetS1, MetS2 and MetS3). The highest calorie (4 Kcal per gram) of fructose led to highest calorie consumption which increased the obesity prevalence and play potential role in metabolic diseases aetiology [38]. The lower calorie in rat chow (2.8 Kcal per gram), tap water (0 Kcal per gram) and PA leaf water extract (2.4 Kcal per gram) led to lower calorie intake in C and PAE. In response to PA extract, the total calorie intake was decreased in MetS3 that reduced the risk of getting metabolic syndrome by reducing lipid deposition formation. The fructose consumption resulted in obesity parameters increment including body weight, body weight gain, BMI and AC. The overweight and obesity measurement were significantly higher in hypertensive compared to normotensive in human study [39]. Previous study reported that treatment with PA leaf water extract (125 mg/kg and 250 mg/kg) in male obese ICR mice treated with HF diet showed an increasing trend of body weight was consistent with this study [40]. Without significance body weight loss, PA supplementation is able to minimise the percentage of body weight gain, BMI and AC in MetS3. The abdominal adipose tissue deposition contributes to central obesity due to excessive unused energy stored as TG due too much calorie intake. Reduced in hypertrophy effects with smaller area of adipocyte without change in whole-body fat suggesting lipid redistribution away from the abdominal area with PA supplementation [40]. The increase in total number of adipocytes and emergence of very small size adipocytes can be seen at the early relapse of progression of obesity [41]. It also can be explained by the animal model surpassing the pre-weight loss following relapse [42, 43]. These effects are most likely caused by high expression of NFκβ p65 that suppressed PPARγ activity which increased energy expenditure by reducing FFA and lipid synthesis resulted in adipose tissue growth and adipogenesis inhibition [44].

This model also showed hypertension, hyperglycaemia and dyslipidaemia. The hypertension may due to rise in cardiac output and total peripheral resistance [7] and was improved with PA supplementation. Interestingly, PA supplementation also helps to reduce fasting plasma glucose in rats without metabolic syndrome induction. This could be possible by glucose metabolism improvement through glycogen synthesis restoration [40]. Although PA supplementation could not decrease TC and LDL, the treatment effectively reduced TG and increased HDL levels. The reduction of hyperlipidaemia could be the result of stimulated lipid storage in fat tissue and suppressed TG concentration in plasma [40]. It is therefore likely that PA leaf water extracts and its major bioactive compounds suppress adipogenesis, thereby allowing excess accumulation of lipids in the liver and the plasma.

Various inflammation markers including NFκβ, TNFα [45, 46], interleukin-6 (IL-6), monocyte chemoattractant protein (MCP-1), visfatin, resistin, leptin [47, 48] and adiponectin [49] have been identified in the metabolic syndrome pathogenesis. In this study, PA supplementation gives neutral effects on NFκβ p65, TNFα, adiponectin and leptin expression. Although NFκβ is the major pro-inflammatory regulator, it is suggested that NFκβ signalling overactivation in adipocytes could prevents obesity and IR [50]. As reported before, the elevated NFκβ p65 protein expression suppressed adipose tissue growth and adipogenesis by PPARγ activity, reduction in FFAs synthesis and activation of lipolysis metabolism resulted in adipocytes size reduction [44]. Contrary to the TNFα plasma concentration, the TNFα was reported to be markedly elevated in metabolic syndrome rat induced with fructose and treatment with ferulic acid suppressed the TNFα production [51]. This is because, TNFα circulating levels initially often found to be low and not significantly elevated because no TNFα systematic release [52]. The elevated leptin plasma concentration was associated with metabolic syndrome regardless previous demographic studies [45, 49]. The reduction in leptin plasma concentration after the PA treatment was consistent to the previous study [53]. Surprisingly, there were no changes in the adiponectin plasma concentration as the levels of adiponectin was expected to be low in the subjects with hypertension and obese [45]. The adiponectin plasma concentration was expected to increase and the result also was consistent as in the previous study, where there were no significance changes in concentration [53]. To support this finding, previous study reported there was no changes in the adiponectin mRNA expressions from adipocytes in high fat diet induced obesity Wistar rat after treatment with the mixture of extract herbal medicines consisting of *Benincasae semen, Laminaria japonica Areschon., Pini Folium, Moli Folium, Citrus aurantium Linn.,* and *Ephedra herb* [54].

In this study, 10 g/100 ml fluid of PA leaf water extract to provide daily dose of 516 mg/kg body weight. This dose corresponds to ~ 5 g/d PA in a 70 kg human according to body surface area comparisons between rats and human [55, 56]. Although the mean daily human intake of PA leaf water extract is not known, the total intake of PA is ~ 5 g/d, with high total phenolic acids (Gallic acid, Cinnamic acid and Ferulic acid) and total flavonoid (Rutin, Epicatechin, Catechin, Kaempferol and Naringin) content. This suggests that the dose of PA leaf water extract used in this study is realistic in human.

## Conclusion

The present study demonstrates for the first time that PA leaf water extracts treatment of metabolic syndrome induced by fructose attenuates or reverse the metabolic changes by reducing body weight gain, BMI, abdominal adipose tissues deposition, adipocytes sizes in abdominal fat tissues, systolic and diastolic BP, FPG, TG and increasing HDL levels with neutral effects on inflammatory biomarkers. Therefore, this suggest beneficial effect of PA in improving metabolic syndrome components. Since the prevalence of metabolic syndrome is increasing in the population worldwide, PA supplementation may serve as complementary dietary strategy to manage metabolic syndrome.

## Data Availability

All the data obtained, and materials analysed in this research are available with the corresponding author on the reasonable request.

## Abbreviations

AC: Abdominal circumference
BMI: Body mass index
BP: Blood pressure
ELISA: Enzyme-linked immunosorbent assay
FFA: Free fatty acids
FLP: Fasting lipid profile
FPG: Fasting plasma glucose
HDL: High density lipoprotein
IDF: International Diabetes Federation
IR: Insulin resistance
LDL: Low density lipoprotein
MCP-1: Monocyte Chemoattractant Protein-1
MetS1: Metabolic syndrome 1
MetS2: Metabolic syndrome 2
MetS3: Metabolic syndrome 3
NCEP ATP III: National Cholesterol Education Program Adult Treatment Program III
NALFD: Non-alcohol fatty liver disease
NFkβ p65: Nuclear Factor Kappa Beta p65
PA: *Pandanus amaryllifolius*
PPARγ: Perixosome Proliferator-Activated Receptor Gamma
RAAS: Renin Angiotensin Aldosterone System
SPSS: Statistic Package for Social Sciences
TC: Total cholesterol
TG: Triglyceride
TNFα: Tumor Necrosis Factor Alpha
WHO: World Health Organization
